# Undetected Circulation of African Swine Fever in Wild Boar, Asia

**DOI:** 10.3201/eid2610.200608

**Published:** 2020-10

**Authors:** Timothée Vergne, Claire Guinat, Dirk U. Pfeiffer

**Affiliations:** National Veterinary School of Toulouse, Toulouse, France (T. Vergne, C. Guinat);; Institut National de Recherche pour l’Agriculture, l’Alimentation et l’Environnement, Toulouse (T. Vergne, C. Guinat);; City University of Hong Kong, Hong Kong, China (D.U. Pfeiffer)

**Keywords:** African swine fever, transmission, wild boar, surveillance, policy, viruses, Sus scrofa, Asia

## Abstract

African swine fever is a growing threat to the livestock industry. We examined data indicating that in most countries in Asia, most notified events were related to farm outbreaks; meanwhile, only a few wild boar cases were reported. We hypothesize the virus circulates unnoticed in wild boar populations in Asia.

African swine fever (ASF) is one of the greatest threats to the livestock industry worldwide. Since 2007, ASF virus (ASFV) has been reported in 34 countries in Europe and Asia (*1*). Some strains of ASF can be associated with case-fatality ratios of almost 100% and with economic damage caused by trade disruptions (*2*). The absence of a safe and effective vaccine and the evolving understanding of the epidemiologic role of wild boar have complicated efforts to control this disease (*3*).

Observations from the Russian Federation, Ukraine, and Romania have suggested that ASFV primarily circulates among small pig farms and spills over into commercial farms occasionally and into wild boar populations regularly (*4*). However, more recent reports from several countries in Europe, including the Baltic states and Belgium, suggest the virus might maintain itself within wild boar populations and occasionally spill over into domestic pig farms. This newly described epidemiologic cycle proposes direct transmission among wild boar and indirect intraspecific transmission through contaminated wild boar carcasses (*4*). This cycle also suggests the persistence of ASFV in wild boar populations even in the context of low wild boar density and despite high death rates caused by the disease (*5*).

In August 2018, ASFV was detected in China, the leading pig producer worldwide. Since then, the virus has also been reported in Mongolia, Vietnam, Cambodia, Hong Kong, North Korea, South Korea, Laos, Myanmar, the Philippines, Timor-Leste, Papua New Guinea, Indonesia, and India (*1*). The large-scale spread of ASFV demonstrates the challenges of controlling the disease in this region, which has a high density of domestic pigs, a high proportion of low biosecurity farms, a widespread practice of feeding pigs with food waste (*6*), and a complex pork trade network involving wet markets and slaughterhouses with poor hygiene (*2*).

Maps of predicted habitat suitability suggest that most areas of East and Southeast Asia are highly suitable for wild boar (*7*). Although information is limited about the spatial distribution of wild boar in Asia, studies suggest that in some regions of China, wild boar density could be similar (*8*) to that of eastern Poland, where ASFV has circulated in wild boar for >5 years (*9*). Reports of crop losses caused by wild boar in China (*10*) indicate that close interactions between wild boar and human activities occur in the region.

The widespread presence of ASFV in pigs in Asia implies regular environmental spillover from the pig supply chain. Therefore, it is highly likely that ASFV is already widely circulating within some wild boar populations in Asia, causing substantial wild boar death. Because life expectancy of ASFV-infected animals is very short, the most effective way to conduct surveillance in boar populations is to monitor reports of infected boar carcasses. Numbers of reported ASFV wild boar cases and farm outbreaks vary by nation. Except for South Korea, which reported 605 infected wild boar carcasses and 17 outbreaks in domestic pig farms from the time of the emergence of the virus in Asia until May 8, 2020, countries in Asia reported a much lower ratio of wild boar cases to farm outbreaks than their counterparts in Europe (Figure) (*1*). In these countries (excluding South Korea), there were only 23 reports of infected wild boar carcasses (in China and Laos only) despite 843 official ASF notifications of farm outbreaks after the virus emerged in Asia in August 2018 (Figure) (*1*).

**Figure:**
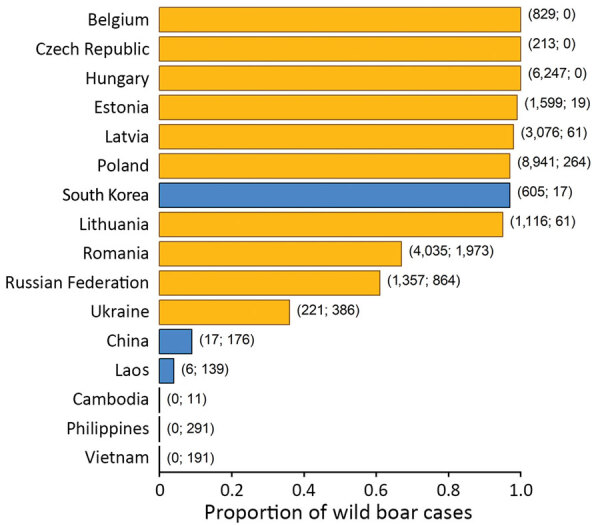
Proportion of wild boar cases out of the total number of reported African swine fever events in the most affected countries in Europe (orange) and Asia (blue). Numbers in parentheses at right side of bars indicate the reported number of wild boar cases and the reported number of outbreaks in farms, from the date of the first reported ASF event in these countries through May 8, 2020 (*1*).

The near absence of notifications of ASFV-infected wild boar cases in Asia highlights shortcomings in surveillance for ASF in wildlife, jeopardizing the success of ASF control policies in the region. We believe this lack of surveillance partly results from government division of responsibilities; in most countries, responsibility for ASF surveillance in livestock belongs to a different government department than that for monitoring wildlife populations (*6*). We argue that long-term success of ASF control in Asia is possible only with risk-based ASF surveillance in wild boar populations by a multisectoral effort of wildlife and agricultural departments. Although surveillance of wild boar is a necessary component of an ASF control strategy, it must be complemented by effective ASF control measures in domestic pigs, such as improved regional coordination, increased resources for surveillance, incentives for farmers to report outbreaks, and enforcement of interventions (*2*). Without these measures, the region might become a major hub of ASFV infection.
